# Moderate-Dose Exercise Protects Against Heart Failure via Apelin/NLRP3 Mediated Inflammation

**DOI:** 10.31083/RCM51174

**Published:** 2026-10-20

**Authors:** Dongli Gao, Maoshuai Zhang, Yuanhao Li, Xuefei Liang, Yue Dong, Xulei Duan, Niannian Ding, Libo Wang, Xuehui Wang

**Affiliations:** ^1^Department of Cardiology, Life Science Research Center, The First Affiliated Hospital of Xinxiang Medical University, 453100 Xinxiang, Henan, China; ^2^Department of Internal Medicine, Henan Yongcheng People’s Hospital, 476600 Yongcheng, Henan, China

**Keywords:** exercise, heart failure, inflammation, metabolism, cardiac rehabilitation

## Abstract

**Background:**

Heart failure (HF) remains a major cause of cardiovascular mortality worldwide, imposing a substantial burden on healthcare systems. Although physical exercise is known to exert cardioprotective effects, its underlying mechanisms are not fully understood.

**Methods:**

We established a post-myocardial infarction (MI) HF mouse model via coronary artery ligation, followed by an 8-week exercise regimen at different intensities. Multi-omics sequencing was performed to identify alterations in inflammatory and metabolic pathways.

**Results:**

Moderate-dose exercise preserved cardiac function and attenuated ventricular remodeling. Metabolomic analysis indicated modulation of lipid metabolism and inflammation via the Apelin pathway. Transcriptomics revealed that the nucleotide-binding oligomerization domain (NOD)-like receptor signaling pathway, which was elevated after MI, was suppressed by moderate-dose exercise. Additionally, Nuclear factor erythroid 2-related factor 2 (Nrf2)—a key transcription factor regulating antioxidant and anti-inflammatory responses—was upregulated in the moderate-exercise group.

**Conclusions:**

Moderate-dose exercise protects against HF in mice by regulating lipid metabolism and inflammatory pathways. These findings support the standardization of exercise therapy for secondary prevention of cardiovascular disease.

## 1. Introduction

Heart failure (HF), a critical endpoint of cardiovascular disease (CVD) progression, has emerged as a global health crisis marked by a persistently rising prevalence [1]. According to the European Society of Cardiology guidelines, HF affects over 2% of the adult population in developed countries, and the five-year survival rate after diagnosis remains below 50% [2]. Consequently, the development of effective therapeutics for HF is urgently required.

Myocardial infarction (MI), typically caused by acute coronary occlusion, leads to ischemic cardiomyocyte death and is the leading cause of HF [3]. This process involves neutrophil/macrophage infiltration and inflammatory cytokine release. Inflammation is an independent risk factor for impaired coronary collateral formation [4]. The NOD-like receptor pyrin domain-containing 3 (NLRP3) inflammasome is a critical mediator of sterile inflammation in cardiovascular pathologies [5,6]. Studies indicate that NLRP3 activity remains low in myocardial tissue within 3 h post-MI, but significantly increases at 3–24 h, driving pathological ventricular remodeling [7,8]. In the heart, NLRP3 activation is primarily regulated by reactive oxygen species (ROS) and dissociation of thioredoxin-interacting protein (TXNIP) from thioredoxins, the accumulation of ROS directly activates NLRP3 and promotes TXNIP dissociation, triggering IL-18/IL-1β release [9].

Nuclear factor erythroid 2-related factor 2 (Nrf2) is the master transcription factor governing cellular antioxidant responses [10]. Under physiological conditions, Nrf2 is sequestered in the cytoplasm by its inhibitor Kelch-like ECH-associated protein 1 (Keap1). Under pathological conditions including ROS accumulation, Nrf2-Keap1 dissociates, enabling Nrf2 nuclear translocation and binding to antioxidant response elements (AREs), thereby upregulating cytoprotective genes such as heme oxygenase-1 (HO-1) [11]. Suppression of the Nrf2/NLRP3 pathway may serve as a potential therapeutic strategy for HF.

Cardiac rehabilitation (CR) is an integrated therapeutic and recovery framework developed in recent years. This multidisciplinary approach encompasses personalized exercise prescriptions, health education, lifestyle modifications, health status assessments, and psychological support [12]. Exercise training, the cornerstone of CR, is a cost-effective and widely accepted intervention that significantly promotes both physical and mental wellbeing [13], possibly by promoting the development of collateral circulation [14]. Moreover, exercise-induced physiological hypertrophy attenuates pathological remodeling post-MI or pressure overload [15]. Although evidence suggests that tailored exercise regimens improve cardiac function and prognosis in patients with HF, its underlying mechanisms remain unclear. Previous studies showed that the activation of Nrf2 by exercise has been demonstrated to attenuate cadmium-induced hepatotoxicity, confirming exercise-mediated Nrf2 activation [16]. Critically, the knockout of endogenous Nrf2 abolishes exercise-induced cardioprotection [17]. Taken together, these findings strongly suggest that Nrf2 activation is a primary mechanism through which exercise confers cardioprotection. Apelin, an adipokine that exists in various molecular isoforms and modulates oxidative stress and angiogenesis, is also implicated in the exercise-induced regulation of lipid metabolism [18]. But its role in HF is not fully understood.

In this study, we demonstrate that moderate-dose exercise preserves cardiac function in experimental HF through Nrf2-dependent suppression of NLRP3. Our work provides a molecular rationale to guide exercise prescription in clinical practice, paving the way for more effective and personalized CR.

## 2. Materials and Methods

### 2.1 Experimental Mouse Model of HF

Male C57BL/6J mice, aged 8–9 weeks and weighing 20–22 g, were obtained from Vital River Laboratory Animal Technology (Beijing, China). The mice were housed under a 12-h light/dark cycle at a controlled temperature of 21 ± 2 °C with free access to food and water. Mice were anesthetized with isoflurane (3–4% concentration, 1.5 L/min gas flow). The MI group underwent permanent ligation of the left anterior descending coronary artery with a 7-0 silk suture. A total of 50 mice were used in this study. Mice that underwent MI surgery were evaluated by echocardiography 2 weeks post-surgery; those with a left ventricular ejection fraction (LVEF) <40% were considered to have successfully developed HF and were included in subsequent experiments. Approximately 20% of MI-operated mice (10 out of 50) did not meet this criterion and were excluded from further analysis. At 2 weeks post-myocardial infarction, mice with an LVEF <40% were randomly divided into different exercise intensity groups. The sample sizes were as follows: Sham (n = 8), MI (n = 8), low exercise doses (Ex_15_) (n = 8), moderate exercise doses (Ex_30_) (n = 8), high exercise doses (Ex_60_) (n = 8). For histological and molecular analyses, subsets of animals (n = 3–8 per group) were randomly selected for each assay. The sham-operated group underwent suture placement without ligation. All mice were euthanized by inhalation of excess isoflurane, followed by cervical dislocation, at the designated experimental endpoints. This euthanasia protocol complies with the guidelines of the Institutional Animal Care and Use Committee and was performed to minimize animal suffering. All the animal experiment protocols were approved by the Laboratory Animal Welfare and Ethics Committee of the First Affiliated Hospital of Xinxiang Medical University (No. EC-023-242), and the animal procedures adhered to the ARRIVE guidelines 2.0. All animal procedures were performed in compliance with the National Institutes of Health (NIH) Guide for the Care and Use of Laboratory Animals.

### 2.2 Exercise Protocol

An additional sham-operated group served as the non-infarcted control. Post-MI HF mice were randomly divided into MI and exercise training groups. All exercise groups underwent a 1-week adaptation period with swimming sessions conducted once daily between 18:00 and 22:00 in a temperature-controlled pool (32–35 °C; 20 cm depth; 80 × 60 × 50 cm). The duration was progressively increased over 7 days as follows: 5 min (day 1), 15 min (day 2), 25 min (day 3), 35 min (day 4), 45 min (day 5), 55 min (day 6), and 60 min (day 7). The mice were then randomized into the following exercise groups: Ex_15_ (low-dose, 15 min/day), Ex_30_ (moderate-dose, 30 min/day), and Ex_60_ (high-dose, 60 min/day). Training occurred 5 days/week for eight weeks.

### 2.3 Echocardiography

Cardiac structure and function were assessed using ultra-high-resolution small-animal ultrasound under continuous anesthesia. Standard two-dimensional echocardiography was performed under anesthesia maintained with 1.5% isoflurane, which resulted in a heart rate range of 450–550 beats/min. The imaging was conducted using a Vevo3100 ultrasound system (Toronto, Ontario, Canada) equipped with a 40-MHz linear array transducer. Cardiac functional assessment was performed using M-mode echocardiography on parasternal long-axis views. All imaging procedures and subsequent analyses were performed by an experienced sonographer blinded to the experimental group assignments.

### 2.4 2,3,5-Triphenyl tetrazolium chloride (TTC) Staining

Hearts were harvested 24 h post-MI, rapidly cooled at –20 °C for 30 min, and sectioned into 1 mm transverse slices. Sections were incubated in a 2% TTC (G3005, Solarbio, Beijing, China) solution (37 °C, dark) followed by 24-hour fixation in 4% paraformaldehyde. Infarct size was quantified to assess model consistency and severity.

### 2.5 Histological Analysis

Cardiac tissues were fixed in 4% paraformaldehyde, dehydrated, embedded in paraffin, and sectioned into 4 μm-thick slices. From each heart, 3–5 sections were prepared and stained. For each section, 5–10 non-overlapping fields were captured under a microscope (20× or 40× magnification) using systematic random sampling. Representative images were selected to reflect the mean values of each group based on quantitative analysis. Myocardial morphology was observed using hematoxylin and eosin (HE) staining (G1120, Solarbio, Beijing, China). Masson’s trichrome (G1340, Solarbio, Beijing, China) and Sirius red staining (G1472, Solarbio, Beijing, China) were used to quantify fibrotic deposition, and wheat germ agglutinin (RL-1022, Solarbio) labeling was used to evaluate myocyte size and cross-sectional area, with subsequent image acquisition using inverted fluorescence microscopy and quantitative analysis performed using ImageJ software (ImageJ 1.52a).

### 2.6 Immunohistochemistry and Immunofluorescence

The sections were dewaxed, rehydrated through an ethanol series, and subjected to citrate-based antigen retrieval. This was followed by permeabilization with 0.3% Triton X-100, blockade of endogenous peroxidase activity with 3% H_2_O_2_, and blocking with 5% BSA in PBS buffer. Subsequently, the sections were incubated overnight at 4 °C with the following primary antibodies (all from China): atrial natriuretic peptide (ANP) (1:2000 dilution, WL02666, Wanleibio), B-type natriuretic peptide (BNP) (1:2000 dilution, WL02126, Wanleibio), cluster of differentiation 31 (CD31) (1:3000 dilution, 280083-1-AP, Sanying), vascular endothelial growth factor A (VEGFA) (1:2000 dilution, WL0009b, Wanleibio), NLRP3 (1:3000 dilution, 30109-1-AP, Sanying), and interleukin 1-beta (IL-1β) (1:3000 dilution, 26048-1-AP, Sanying). Following incubation with appropriate secondary antibodies, the sections were counterstained with hematoxylin. Images were acquired using an inverted fluorescence microscope (Leica, Germany). The positively stained areas were quantified using ImageJ software (v1.52a).

### 2.7 Western Blotting

Cardiac tissue lysates were quantified via bicinchoninic acid (BCA) assay, separated by sodium dodecyl sulfate‑polyacrylamide gel electrophoresis (SDS-PAGE), and transferred to polyvinylidene difluoride (PVDF) membranes. The membranes were then incubated overnight at 4 °C with the following primary antibodies: anti-alpha-smooth muscle actin (α-SMA) (1:3000 dilution, 80008-1-RR, Sanying), anti-NLRP3 (1:3000 dilution, 30109-1-AP, Sanying), anti-IL-1β (1:3000 dilution, 26048-1-AP, Sanying), anti-Nrf2 (1:1000 dilution, T55136, Abmart), anti-HO-1 (1:1000 dilution, T55113, Abmart) and anti-Apelin (1:1000 dilution, ER65472, Huabio). Protein bands were detected using the Amersham Imager 800 system (Cytiva Life Sciences), and protein expression levels were analyzed using ImageJ software (v1.52a).

### 2.8 Transcriptomics and Metabolomics

Cardiac tissues and serum samples from the sham, MI, and moderate-dose exercise groups (n = 4 per group) underwent eukaryotic reference-based RNA sequencing, which was performed by Wuhan Frasergen Bioinformatics Co. Ltd. For Kyoto Encyclopedia of Genes and Genomes (KEGG) pathway enrichment analysis, differentially expressed genes (DEGs) were identified using the DESeq2 package with thresholds of |log_2_FC| >1 and false discovery rate (FDR) <0.05. KEGG enrichment analysis was performed using the clusterProfiler package (R version 4.0) and the OmicStudio platform. Enriched pathways were considered significant when the FDR-adjusted *p*-value (Q-value) was <0.05. Enrichment analysis was performed on DEGs identified from cardiac tissue RNA sequencing, thereby directly reflecting cardiac metabolic and inflammatory pathways.

### 2.9 Statistical Analysis

All data are presented as mean ± standard deviation (SD). Prior to analysis, the normality of data distribution was assessed using the Shapiro-Wilk test, and homogeneity of variances was evaluated using Levene’s test. For all datasets presented, these assumptions were satisfied (*p* > 0.05). Comparisons between multiple groups were therefore performed using one-way ANOVA followed by Tukey’s post-hoc test to isolate specific differences. Statistical significance was set at *p* < 0.05. No samples were excluded as outliers in any analysis. All animals that met the inclusion criteria (LVEF <40% post-MI) were included. For RNA sequencing and metabolomics, all four samples per group were included, and no samples were excluded based on outlier criteria.

## 3. Results

### 3.1 Exercises Increase Cardiac Function in Mice With HF

A mouse model of HF post-MI was successfully established (Fig. 1A). HF mice underwent a one-week swimming acclimation period two weeks after MI surgery. In the fourth week, mice were divided into three groups for swimming training at different doses. The exercise protocol was conducted 5 days per week over a period of 7 weeks (The total training duration was 8 weeks. During the first week, the mice underwent adaptive swimming. For the remaining 7 weeks, the mice swam for the corresponding duration). Cardiac function was assessed using M-mode echocardiography (Fig. 1B). HF mice exhibited impaired cardiac function compared to sham mice, with signiﬁcantly reduced LVEF, left ventricular fractional shortening (LVFS), and left ventricle stroke volume (LVSV) (Fig. 1D,E,H). Notably, mice in the moderate-dose exercise group exhibited improved cardiac function, with better preserved LVEF, LVFS, and LVSV (Fig. 1D,E,H). The heart rates were similar between the groups (Fig. 1C). Furthermore, mice in the MI group exhibited an increased left ventricular internal dimension at end-diastole (LVIDd) and end-systole (LVIDs) compared to the sham group, which was signiﬁcantly attenuated after moderate-dose exercise (Fig. 1F,G). Moreover, the MI group exhibited an increased heart weight-to-tibia length ratio (HW/TL) compared to the sham group, which was signiﬁcantly attenuated after moderate-dose exercise (Fig. 1I). These results suggest that moderate-dose exercise can significantly protect the heart from established MI.

**Fig. 1.**
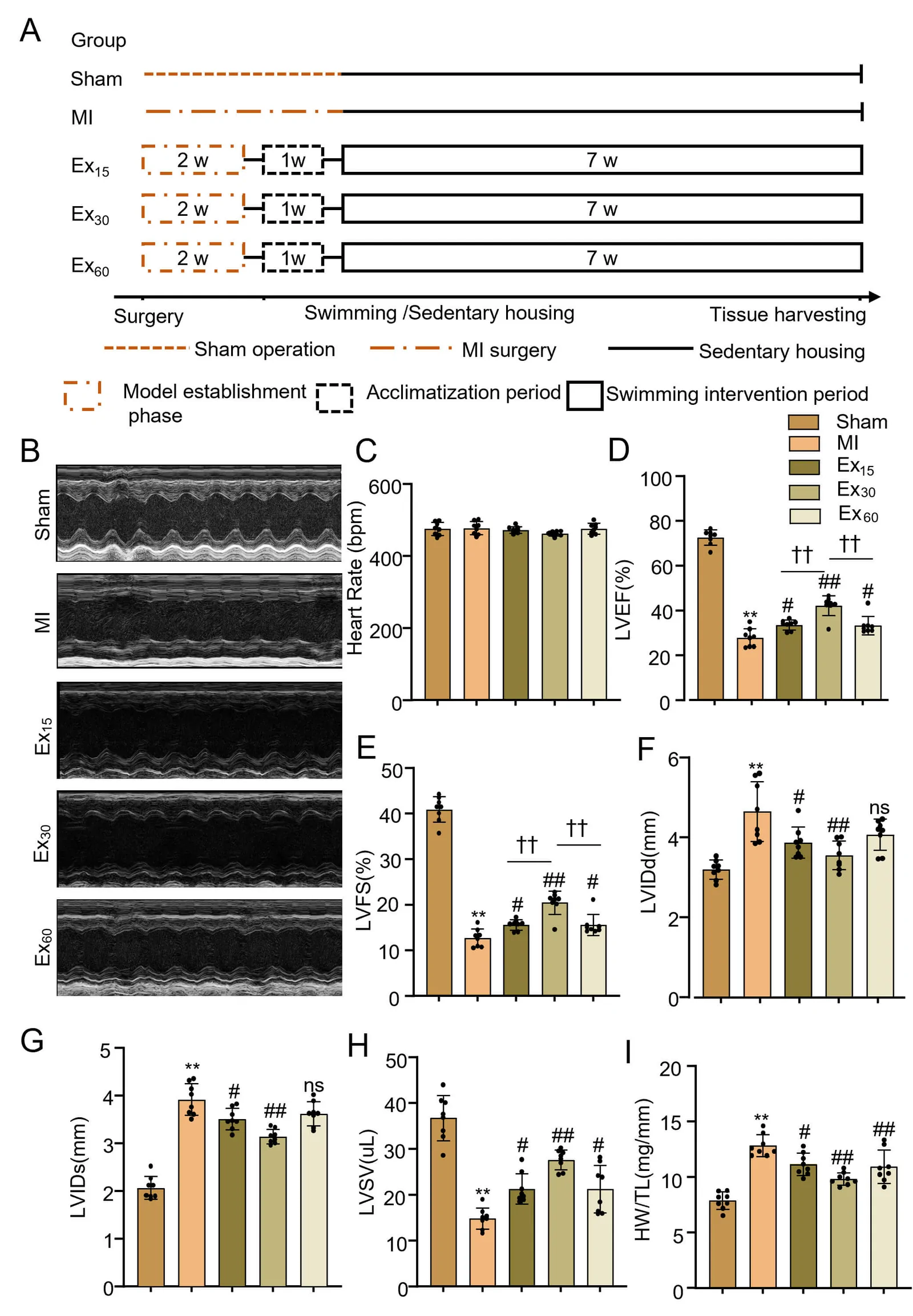
**Exercise increases cardiac function in mice with HF**. (A) Exercise training protocol initiated 2 weeks after MI surgery. (B) Representative echocardiographic images of M-mode in mice of different groups after 8 weeks of exercise training. (C) Heart rate. (D) Left ventricular ejection fraction (LVEF). (E) Left ventricular fractional shortening (LVFS). (F) Left ventricular internal dimension at end-diastole (LVIDd) and (G) at end-systole (LVIDs). (H) Left ventricular stroke volume (LVSV). (I) Heart weight to tibia length ratio (HW/TL). **p < 0.01 compared with sham group; #*p* < 0.05, ##*p* < 0.01 compared with MI group; ^††^*p* < 0.01 compared with Ex_30_ group; One-way ANOVA followed by Tukey’s post hoc test. All data are mean ± standard deviation (SD). HF, heart failure; MI, myocardial infarction; ns, not significant.

### 3.2 Moderate-Dose Exercise Alleviates Ventricular Remodeling in Mice With HF

Ventricular remodeling is an important pathological mechanism involved in the development and progression of HF after MI. Next, we examined whether exercise protects against ventricular remodeling. The MI group exhibited significant cardiac enlargement compared with the sham group. Moderate-dose exercise mice showed a decreased heart size (Fig. 2A). To assess myocardial fibrosis, we performed HE staining, Sirius Red staining, and Masson’s trichrome staining. The MI group showed signiﬁcantly aggravated cardiac ﬁbrosis compared with the sham group, as evidenced by the increased area of fibrosis (Fig. 2B,E–H). Immunoblot analysis further conﬁrmed the elevated levels of α-SMA, a myocardial ﬁbrosis marker, in HF mouse hearts (Fig. 2I,J). Strikingly, moderate-dose exercise signiﬁcantly reduced cardiac border zone ﬁbrosis and α-SMA expression in MI mice. Wheat germ agglutinin (WGA) staining demonstrated a significant increase in the cardiomyocyte cross-sectional area in the MI group compared with that in the sham group. The low- and moderate-dose exercise groups showed a marked reduction in the cardiomyocyte cross-sectional area, suggesting regression of pathological hypertrophy (Fig. 2C,D). These findings demonstrate that moderate-dose exercise exerts a beneficial effect on ventricular remodeling compared to the MI group, likely by inhibiting myocardial ﬁbrosis and hypertrophy in HF mice.

**Fig. 2.**
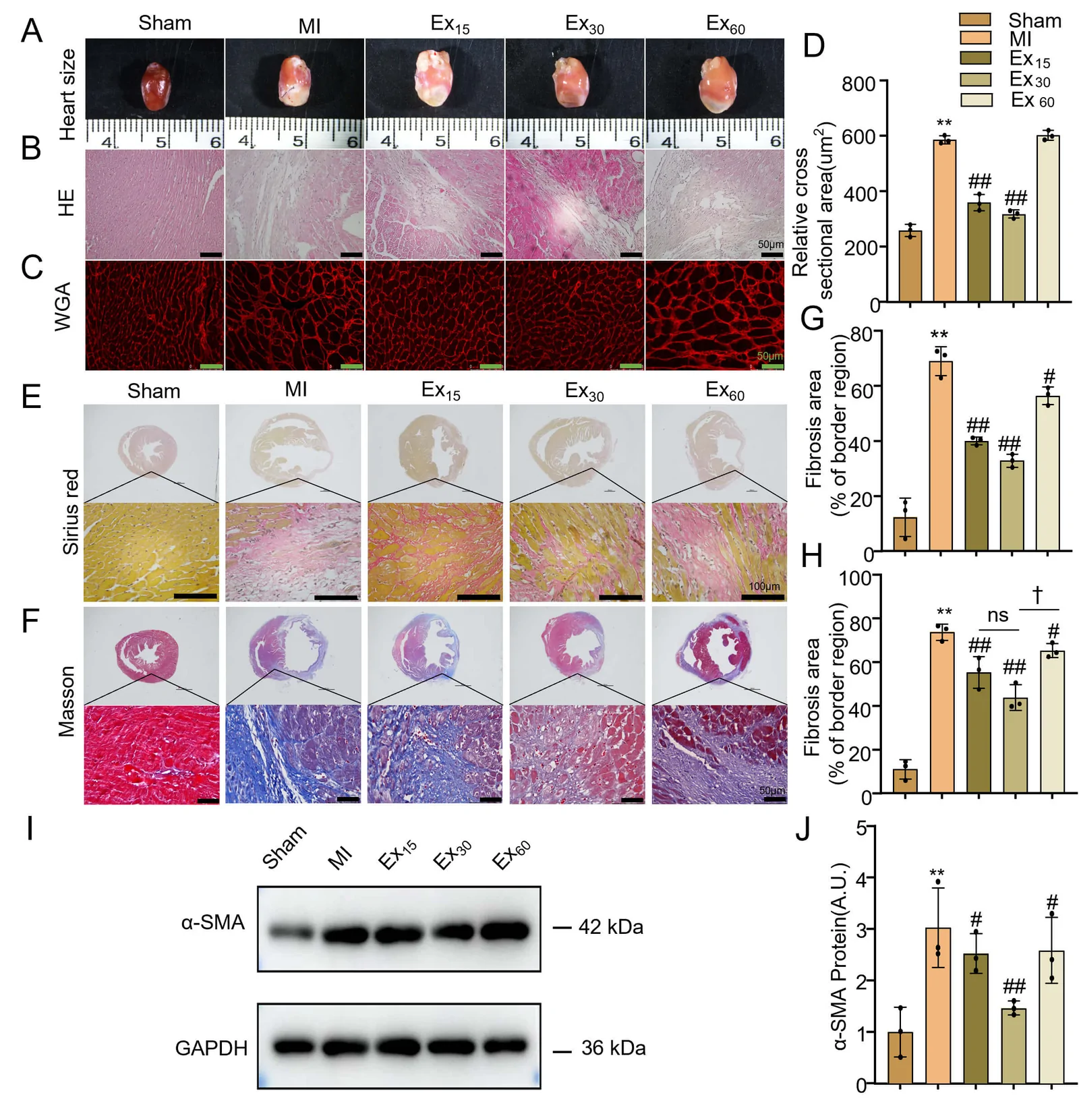
**Moderate-dose exercise alleviates ventricular remodeling in mice with HF**. (A) Gross morphological changes of hearts after 8-week swimming training. (B) Representative HE-stained cardiac sections of different groups, Scale bars: 50 μm. (C,D) Representative sections for cardiomyocyte area (WGA-stained) and its mean data, Scale bars: 50 μm. (E,G) Interstitial fibrosis (Picrosirius red-stained) and its mean data, Scale bars: 100 μm. (F,H) Masson’s trichrome staining and its mean data, Scale bars: 50 μm. (I,J) Protein levels of α-SMA in hearts of mice from different groups and its mean data of protein immunoblots, n = 3/group. **p < 0.01 compared with sham group; #*p* < 0.05, ##*p* < 0.01 compared with MI group; ^†^*p* < 0.05 compared with Ex_30_ group; One-way ANOVA followed by Tukey’s post hoc test. All data are mean ± SD. α-SMA, alpha-smooth muscle actin; HF, heart failure; ns, not significant; WGA, wheat germ agglutinin.

### 3.3 HF Biomarkers Were Decreased in HF Mice Treated With Moderate-Dose Exercise

To further evaluate the effect of moderate-dose exercise on HF biomarkers, we assessed the expression of BNP and ANP by immunohistochemistry. BNP and ANP expression were significantly upregulated in the MI group compared with the sham group. This upregulation was diminished by low-, moderate-, and high-dose exercise (Fig. 3A–C). Notably, moderate-dose exercise resulted in the most significant reduction in BNP and ANP levels. These results suggest that moderate-dose exercise confers the most significant cardioprotective effects.

**Fig. 3.**
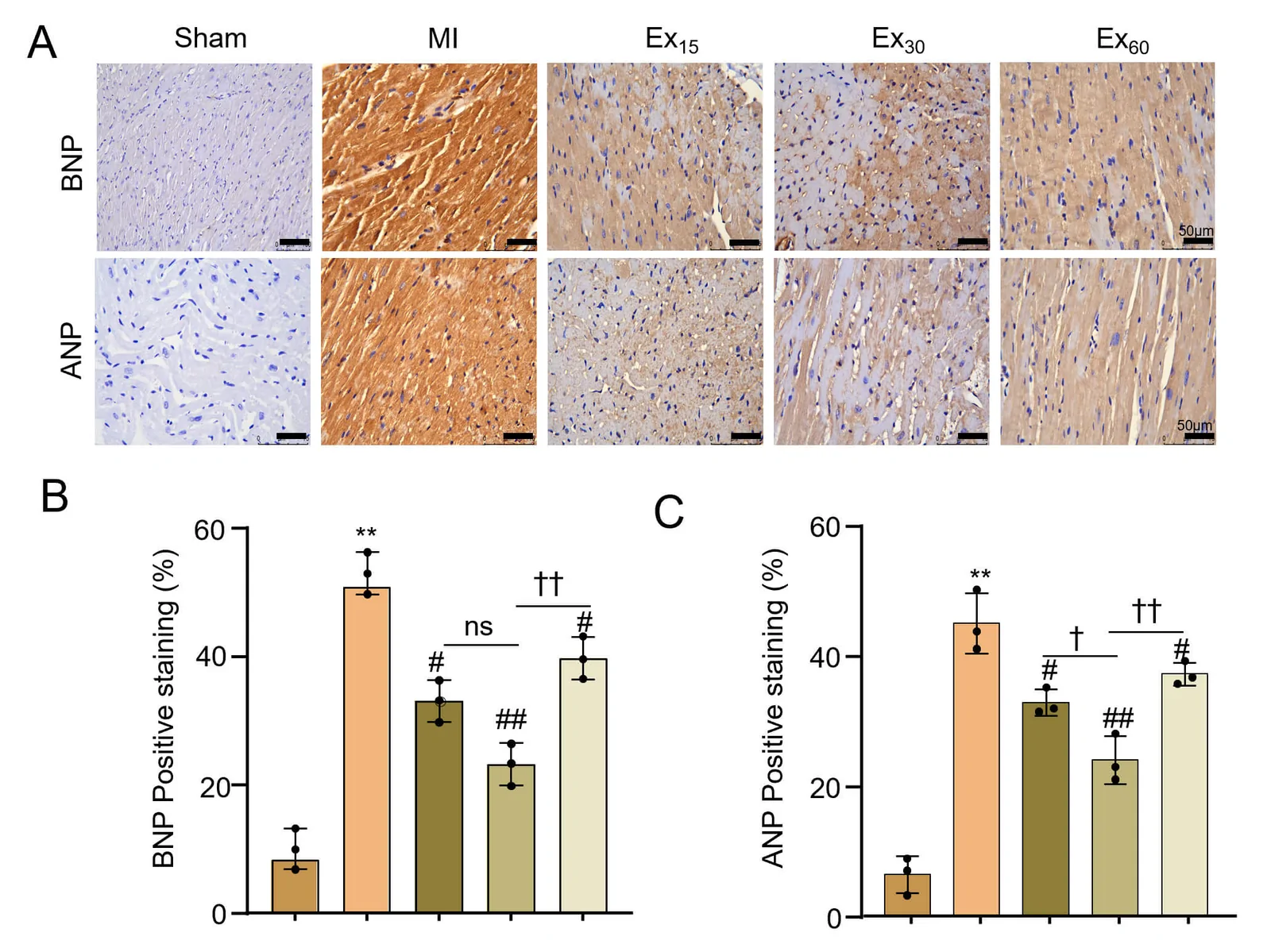
**HF biomarkers were decreased in HF mice treated with moderate-dose exercise**. (A) Immunohistochemical analysis of BNP and ANP with the heart tissues in HF mice from different groups, Scale bars: 50 μm. (B,C) Quantitation of BNP and ANP positive staining, n = 3/group. **p < 0.01 compared with sham group; #*p* < 0.05, ##*p* < 0.01 compared with MI group; ^†^*p* < 0.05, ^††^*p* < 0.01 compared with Ex_30_ group; One-way ANOVA followed by Tukey’s post hoc test. All data are mean ± SD. ANP, atrial natriuretic peptide; BNP, B‑type natriuretic peptide; ns, not significant.

### 3.4 Moderate-Dose Exercise Increases the Collateral Circulation in the Infarction Areas of the Heart in HF Mice

To investigate whether collateral circulation plays a role in exercise-induced cardioprotection, we used immunofluorescence to detect the expression of CD31 and VEGFA in HF mice from different groups. Immunofluorescence revealed that the expression of CD31 and VEGFA was downregulated in the hearts of HF mice. This downregulation was reversed by moderate-dose exercise (Fig. 4A,B). These findings suggest that the cardioprotective effect of moderate-dose exercise may be mediated, at least in part, by the promotion of angiogenesis.

**Fig. 4.**
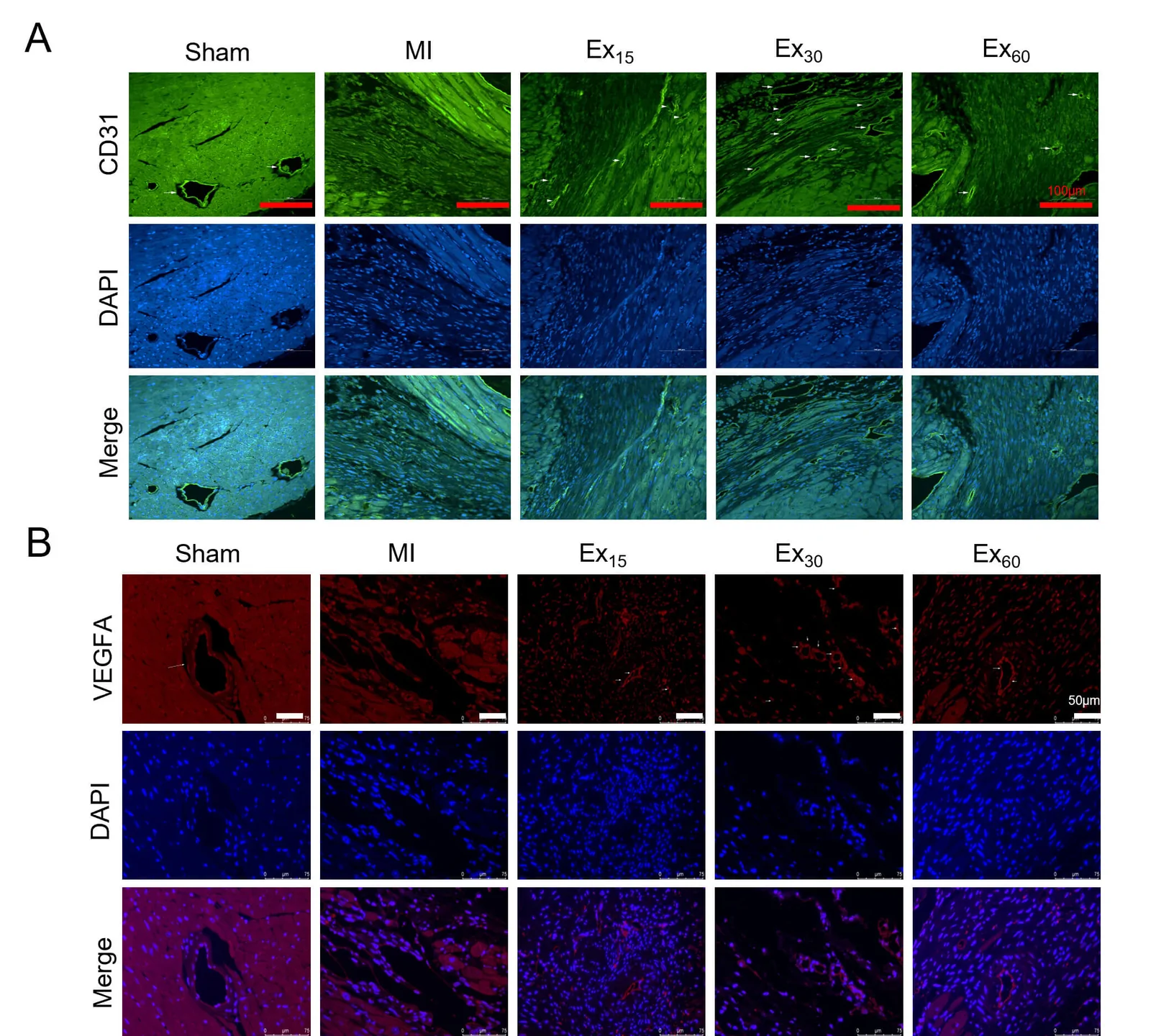
**Moderate-dose exercise increases the collateral circulation in the infarction areas of the heart in HF mice**. (A,B) Immunofluorescence staining to visualize the localization of CD31 and VEGFA in the myocardial infarction (MI) border zone of mouse hearts from different groups. Scale bars: 50 μm (white bars), 100 μm (red bars). CD31, Cluster of Differentiation 31; VEGFA, Vascular Endothelial Growth Factor A.

### 3.5 Moderate-Dose Exercise Modulates Lipid Metabolism via Apelin Pathway

To investigate the mechanisms by which exercise improves HF, we performed serum metabolomic analysis. Results showed significant differences in lipid metabolites or the Apelin signaling pathway among the Sham, MI, and moderate-dose exercise groups in KEGG pathway analysis, with statistical significance (Fig. 5A–D). Immunoblot analysis further conﬁrmed elevated levels of Apelin in the moderate-dose exercise group (Fig. 5E,F). Therefore, moderate-dose exercise protects against HF by modulating cardiac lipid metabolism, which contributes to its antioxidant and anti-inflammatory effects.

**Fig. 5.**
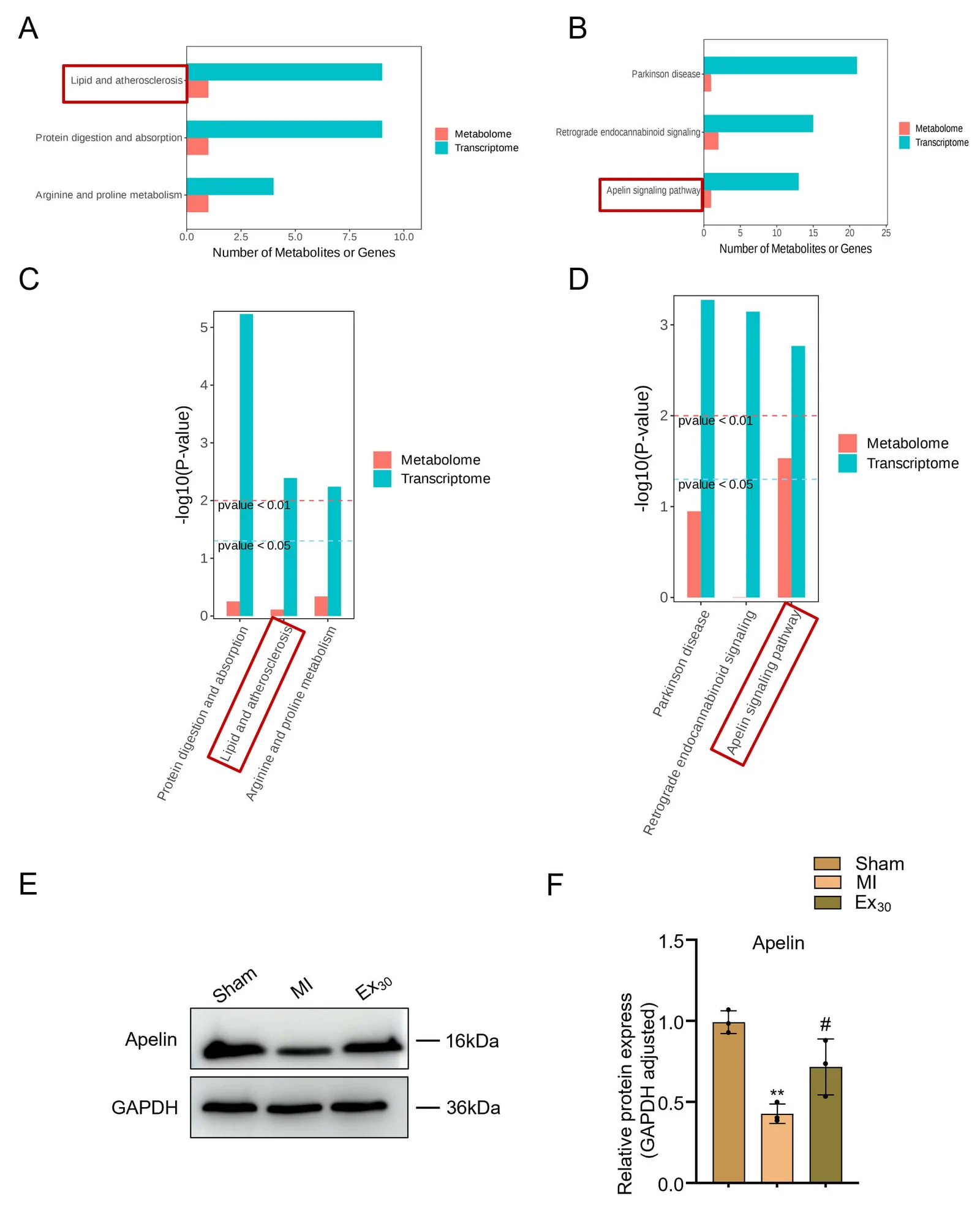
**Moderate-dose exercise modulates lipid metabolism via Apelin pathway**. (A,B) KEGG pathway enrichment analysis of differential molecules was performed for the sham vs. MI (A) and MI vs. moderate-dose exercise group (B) comparisons, respectively, showing the number of altered molecules within three core pathways at both the metabolome and transcriptome levels. (C,D) Bar charts display the *p*-values of KEGG enrichment for three core pathways at the metabolome and transcriptome levels in the sham vs. MI (C) and MI vs. moderate-dose exercise group (D) comparisons, respectively. (E,F) Protein levels of Apelin in the hearts of mice from different groups and their mean data of protein immunoblots, n = 3/group. **p < 0.01 compared with sham group; #*p* < 0.05 compared with MI group; One-way ANOVA followed by Tukey’s post hoc test. All data are mean ± standard deviation (SD).

### 3.6 Moderate-Dose Exercise Modulates Lipid Metabolism and Inflammation in Mice With HF

Prior evidence indicates that Apelin modulates the Nrf2/NLRP3 signaling pathway, which is critically involved in antioxidant and anti-inflammatory responses. Specifically, Apelin activates Nrf2, thereby mediating its anti-inflammatory effects. To further elucidate the role of inflammation in HF, we performed cardiac transcriptomic sequencing following MI, aiming to demonstrate the involvement of the NLRP3 inflammasome in post-MI HF. Eukaryotic reference-based RNA sequencing was performed on cardiac tissues from the sham, MI, and moderate-dose exercise groups to elucidate underlying mechanisms. First, an inter-sample correlation assessment (Fig. 6A) confirmed the strong intra-group reproducibility and clear separation between groups. Differential gene expression (DGE) analysis identified 500 DEGs between the sham and MI groups. Furthermore, a comparison of the MI group with the moderate-dose exercise group revealed 1840 DEGs (Fig. 6B). Further analysis of the top 20 enriched pathways (Fig. 6C,D) revealed significant enrichment for several pathways, most notably the NOD-like receptor signaling pathway. This canonical inflammatory pathway has been previously implicated in pathological ventricular remodeling and collateral circulation.

**Fig. 6.**
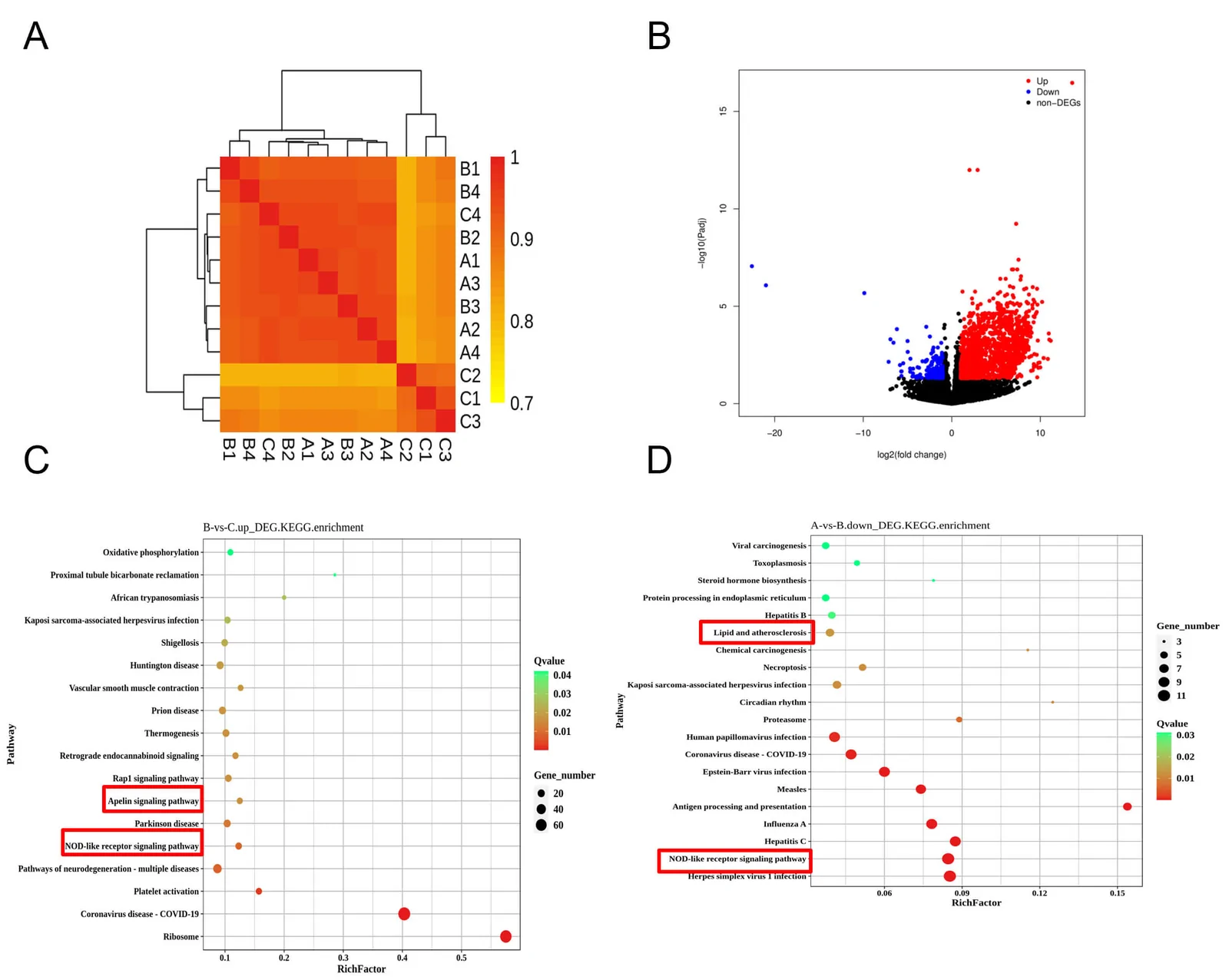
**Moderate-dose exercise modulates lipid metabolism and inflammation in mice with HF**. (A) Heatmap of intra-group sample correlation analysis results. Colored bars from yellow to red represent the correlation coefficient from low to high. A correlation coefficient closer to 1 indicates higher similarity in expression patterns between samples. (B) Volcano plot analysis of 1840 DEGs between the 2 groups. Each dot represents a gene detected in both groups. Red and blue dots indicate genes with significantly upregulated and downregulated expression, respectively. Black dots represent genes that did not show significant differential expression between the MI vs Ex30 (filter criteria: FDR <0.05, |log_2_FC| >1). (C,D) The horizontal axis of the bubble plot represents the Rich Factor. Bubble plots show the KEGG pathway enrichment of down-regulated genes in the sham vs. MI group (D) comparison of up-regulated genes in the MI vs. moderate-dose exercise group (C), respectively; larger bubbles indicate a greater number of genes. Additionally, the color of the bubbles represents the significance of enrichment, measured by the Q-value, which is a *p*-value adjusted for FDR. DEGs, differentially expressed genes; FDR, false discovery rate; KEGG, Kyoto Encyclopedia of Genes and Genomes.

### 3.7 Moderate-Dose Exercise Suppresses Inflammation in HF Mice Via the Nrf2 Pathway

We examined whether suppression of NLRP3 is involved in moderate-dose exercise-induced cardioprotection. Immunofluorescence analysis revealed that the MI group exhibited increased NLRP3 and IL-1β expression compared to the sham group (Fig. 7A–D). Immunoblot analysis further conﬁrmed elevated levels of NLRP3 and IL-1β in HF mouse hearts (Fig. 7E–G). Notably, moderate-dose exercise signiﬁcantly reduced NLRP3 and IL-1β expression in HF mice. To determine whether Nrf2 is involved in moderate-dose exercise-induced cardioprotection, we assessed Nrf2 and HO-1 expression by immunoblot analysis. The results showed that the expression of Nrf2 and HO-1 was upregulated in the MI group compared to the sham group. Notably, moderate-dose exercise further enhanced this upregulation (Fig. 7H–J). These findings indicate that exercise likely ameliorates cardiac dysfunction in HF by exerting anti-inflammatory effects that are associated with the activation of the Nrf2 pathway.

**Fig. 7.**
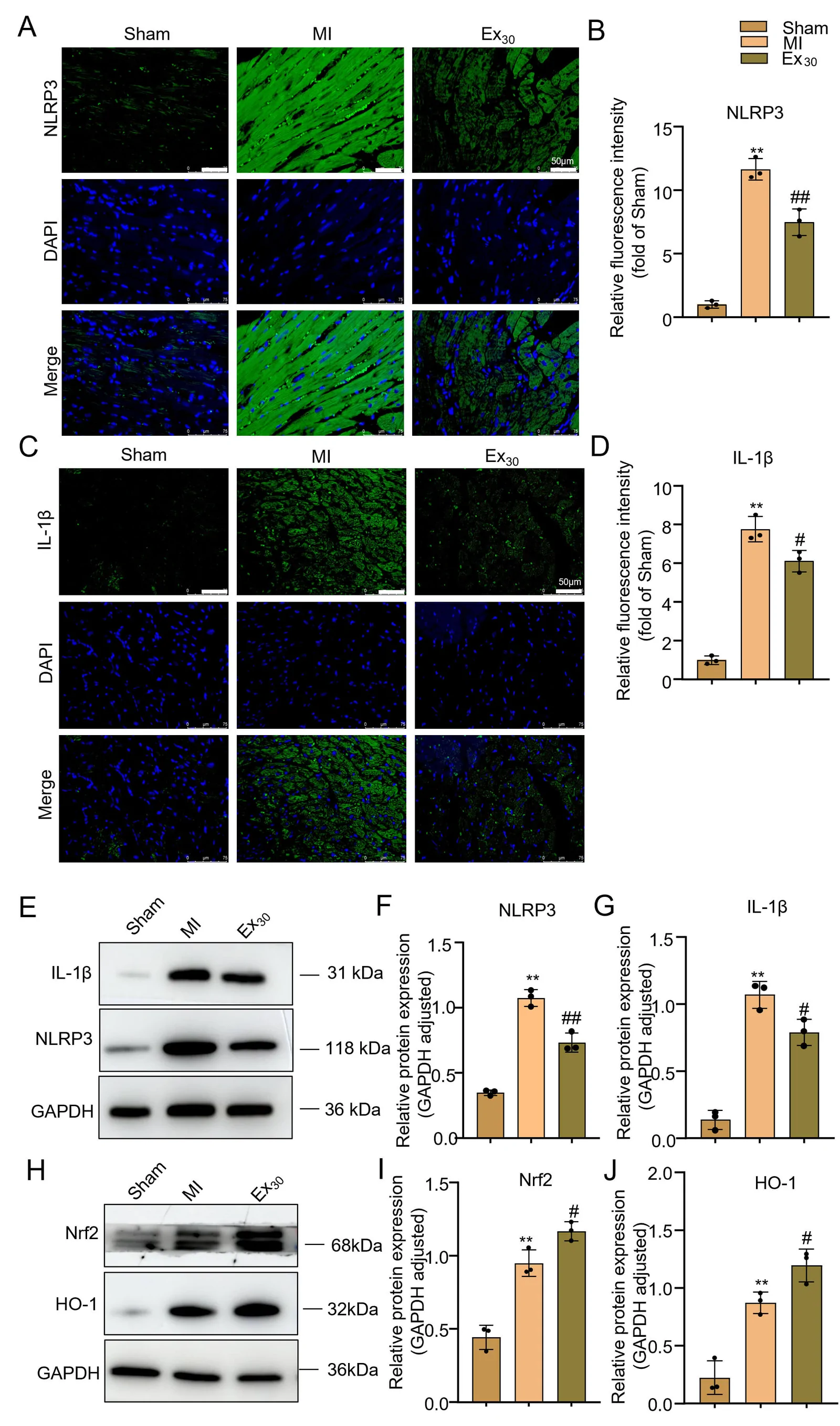
**Moderate-dose exercise suppresses inflammation in HF mice via the Nrf2 pathway**. (A,B) Immunofluorescence staining of NLRP3 in the hearts of mice and quantitation of its positive staining. (C,D) Immunofluorescence staining of IL-1β in the hearts of mice and quantitation of its positive staining. Scale bars: 50 μm; n = 3/group. (E–G) Protein levels of NLRP3 and IL-1β in hearts of mice from different groups and their mean data of protein immunoblots, n = 3/group. (H–J) Protein levels of Nrf2 and HO-1 in the hearts of mice from different groups and their mean data of protein immunoblots, n = 3/group. **p < 0.01 compared with sham group; #*p* < 0.05, ##*p* < 0.01 compared with the MI group; One-way ANOVA followed by Tukey’s post hoc test. All data are mean ± SD. Nrf2, Nuclear factor erythroid 2-related factor 2; NLRP3, NOD-like receptor pyrin domain-containing 3; IL-1β, interleukin 1-beta; HO-1, heme oxygenase-1.

## 4. Discussion

HF is a complex clinical syndrome representing the ultimate manifestation of various cardiovascular diseases, characterized by impaired ventricular systolic and/or diastolic function, along with associated clinical signs and symptoms [19]. It imposes a substantial societal burden and remains a major therapeutic challenge in cardiology.

Exercise, as the cornerstone of CR, serves as a critical adjuvant therapy that confers cardioprotection through anti-inflammatory, antioxidant, and anti-apoptotic effects. Nyawo et al. [20] demonstrated that moderate-dose exercise reduces vascular inflammation and oxidative stress. Moreover, exercise has been shown to ameliorate myocardial ischemia by promoting coronary collateral circulation, leading to increased blood flow, improved cardiac functional reserve (10–30%), and a higher ischemic threshold [21]. Consistent with this, previous narrative reviews emphasize that exercise protects cardiac function by improving ventricular remodeling post-MI [22]. However, a meta-analysis by Slimani et al. [23] found that exercise frequency and duration did not significantly improve certain cardiac function indicators such as LVEF. Previous studies have reported nuanced effects of exercise duration on post-MI HF. Lu et al. [24] showed that prolonged swimming (60 min/day for eight weeks) improved cardiac function, attenuated ventricular remodeling, and reduced myocardial inflammation in mice. In contrast, Tan et al. [25] revealed a paradoxical effect: while brief swimming (15 min/day, eight weeks) enhanced cardiac function in late-stage post-MI HF, it offered limited anti-remodeling benefits. Prolonged swimming under their conditions exacerbated ventricular remodeling, increased fibrosis, and impaired cardiac function. These discrepancies likely stem from methodological variations, including differences in animal strains and the timing of exercise initiation relative to MI induction. Therefore, clarifying the effects of different exercise doses on HF progression is imperative.

In our study, compared with sham controls, MI mice exhibited cardiac hypertrophy and myocardial fibrosis. Low-to-moderatedose exercise, particularly moderate-dose exercise, significantly attenuated post-MI ventricular remodeling. In contrast, high-dose exercise did not confer significant benefits in improving ventricular remodeling. Compensatory coronary collateralization occurs post-MI to salvage ischemic myocardium, consistent with a previous review concluding that exercise enhances collateral circulation [26]. Immunofluorescence analysis of border zones in MI mice revealed minimal collateral circulation in the MI group, modest neovascularization with low- and high-dose exercise, and substantial angiogenesis in the moderate-dose group. Collectively, our comparison of different exercise intensities highlights the distinct superiority of moderate-dose exercise in providing cardioprotection, specifically through ameliorating ventricular remodeling and promoting collateral circulation. The selection of 15, 30, and 60 minutes of daily swimming was designed to model low, moderate, and high exercise doses in mice, based on previously established protocols [25,27]. The 30-minute regimen, which conferred optimal cardioprotection in our study, aligns with current clinical recommendations for 150 minutes of moderate-intensity aerobic activity per week, supporting its translational relevance.

To elucidate the cardioprotective mechanisms of moderate-dose exercise, metabolomic screening identified Apelin as a molecule of interest. Subsequent RNA sequencing of myocardial tissue revealed significant enrichment of the NOD-like receptor signaling pathway in KEGG analysis. This pathway is a well-documented inflammatory cascade involved in pathological ventricular remodeling [28,29], strongly implying its role in exercise-mediated cardioprotection. Within this pathway, the NLRP3 inflammasome is most closely associated with cardiovascular pathology.

NLRP3 activation plays a mechanistic role in cardiovascular disease. For instance, in type 2 diabetic rats with cardiovascular complications, NLRP3 hyperactivation exacerbates pathological ventricular remodeling, an effect reversed by NLRP3 silencing [30]. Toldo et al. [7] demonstrated that NLRP3 inflammasome activation during ischemia promotes myocardial fibrosis and cardiomyocyte hypertrophy. Moreover, an elevated systemic immune-inflammation index is linked to impaired coronary collateral circulation [31]. Consequently, NLRP3 inhibition represents a promising therapeutic strategy. Evidence suggests that MCC950 attenuates pressure overload–induced cardiac dysfunction by suppressing NLRP3, thereby reducing myocardial inflammation and collagen deposition [32]. Nrf2, a pivotal intracellular antioxidant transcription factor, effectively suppresses NLRP3 expression, exerting antioxidative and anti-inflammatory effects. For example, resveratrol enhances Nrf2 signaling, upregulating downstream antioxidants such as HO-1 and NAD(P)H dehydrogenase quinone 1 (NQO1), leading to attenuated oxidative stress and inhibition of the TXNIP/NLRP3/caspase-1 inflammasome [33]. Mechanistically, activated Nrf2 translocates to the nucleus, reduces ROS levels, suppresses NLRP3 inflammasome assembly, and mitigates pyroptosis and inflammation.

Exercise offers notable advantages as a non-pharmacological, noninvasive intervention. Substantial evidence confirms that exercise activates Nrf2, conferring protection to joints, neural tissue, liver, and kidneys [16,34,35,36]. Our experimental data indicate a reciprocal relationship: expression of NLRP3 and its downstream effector IL-1β was markedly elevated in failing hearts but robustly suppressed following moderate-dose exercise. This anti-inflammatory effect was paralleled by significant upregulation of Nrf2. These coordinated changes strongly suggest that mitigation of NLRP3-driven inflammation is a pivotal mechanism through which exercise protects the heart, and that Nrf2 activation plays an instrumental role. Interestingly, Apelin—a peptide hormone with established antioxidant and anti-inflammatory properties of Apelin are now supported by [37]. Our multi-omics data consistently showed induction of Apelin in response to moderate-dose exercise. Therefore, we propose a coherent mechanistic cascade: moderate-dose exercise upregulates cardiac Apelin, which activates the Nrf2 antioxidant pathway. Activated Nrf2 then transcriptionally represses the NLRP3 inflammasome, leading to diminished inflammation, attenuated ventricular remodeling, and ultimately improved cardiac function in post-MI HF.

### Limitation

First of all, our findings indicate a clear association between moderate-dose exercise and the upregulation of Apelin and Nrf2 alongside suppression of NLRP3 and IL-1β, we acknowledge that causal relationships have not been definitively established in the current study. Nonetheless, convergent evidence from previous studies supports the proposed axis: exercise-induced Apelin has been shown to activate Nrf2 signaling [18], and Nrf2 activation is a well-established negative regulator of the NLRP3 inflammasome [38]. Future studies employing Apelin receptor antagonists, Nrf2 knockout mice, or NLRP3-specific inhibitors are warranted to validate this mechanistic cascade. We also recognize that the cardioprotective effects of moderate-dose exercise are likely multifactorial, involving contributions from enhanced angiogenesis, improved metabolic homeostasis, and broader anti-inflammatory responses beyond the Apelin/Nrf2/NLRP3 axis. In contrast to the beneficial effects of moderate-dose exercise, high-dose exercise (60 min/day) did not improve ventricular remodeling and was associated with increased fibrosis [39,40]. This observation is consistent with previous studies reporting that excessive exercise may exacerbate oxidative stress and sympathetic activation, potentially leading to adverse remodeling in the compromised post-MI heart.

The translational relevance of our findings is supported by recent large-scale cohort studies demonstrating that higher exercise capacity is associated with significantly lower risks of HF in humans [41,42,43]. Notably, the dose-response relationship observed in our murine model—where moderate, but not excessive, exercise conferred optimal cardioprotection—parallels human evidence suggesting that moderate-intensity exercise provides maximal cardiovascular benefit, whereas extreme volumes may attenuate these protective effects. While the multi-omics data were derived from a relatively small sample size (n = 4 per group) and should be interpreted as exploratory, the key pathways identified—particularly the NOD-like receptor signaling pathway and Apelin-related lipid metabolism—were consistently validated using independent techniques and larger sample sizes, supporting the robustness of our conclusions. We acknowledge that serum metabolites reflect systemic metabolism, which may not directly mirror cardiac-specific changes. However, given the established role of Apelin as an adipokine that modulates both systemic and cardiac lipid metabolism, and the parallel changes observed in cardiac transcriptomics (NOD-like receptor pathway) and serum metabolomics, these datasets provide complementary insights into the cardioprotective mechanisms of exercise.

## 5. Conclusion

In conclusion, our study indicates that moderate-dose exercise effectively ameliorates cardiac dysfunction in post-MI HF, primarily through attenuating adverse ventricular remodeling and enhancing coronary collateral circulation. We further elucidate a novel molecular mechanism underlying these benefits: moderate-dose exercise upregulates the adipokine Apelin, which activates the Nrf2 antioxidant pathway. This activation leads to transcriptional repression of the NLRP3 inflammasome, thereby attenuating maladaptive inflammation and mediating cardioprotection. These insights provide a mechanistic rationale to guide the optimization of exercise prescription in clinical practice. Our work underscores the translational potential of prescribing precise, moderate-dose exercise regimens as a safe and effective adjunctive strategy for managing HF.

## Data Availability

The raw data for this article are made available upon request from the corresponding authors.

## Abbreviations

HF, heart failure; MI, myocardial infarction; CVD, cardiovascular disease; CR, cardiac rehabilitation; Nrf2, Nuclear factor erythroid 2-related factor 2; HO-1, heme oxygenase-1; NLRP3, NOD-like receptor pyrin domain-containing 3; LVEF, left ventricular ejection fraction; LVFS, left ventricular fractional shortening; LVIDs, left ventricular internal dimension at end-systole; LVIDd, left ventricular internal dimension at end-diastole; HW/TL, heart weight to tibia length ratio; ANP, atrial natriuretic peptide; BNP, B-type natriuretic peptide; CD31, cluster of differentiation 31; VEGFA, vascular endothelial growth factor A; α-SMA, alpha-smooth muscle actin; DGE, differential gene expression; FDR, false discovery rate.
